# Distribution and identification of *Mycobacterium tuberculosis* lineage in Kashgar prefecture

**DOI:** 10.1186/s12879-022-07307-4

**Published:** 2022-03-30

**Authors:** Ai-Min Xu, Chuan-Jiang He, Xiang Cheng, AniKiz Abuduaini, Zureguli Tuerxun, Yin-Zhong Sha, Aihemaitijiang Kaisaier, Hong-Mei Peng, Ya-Hui Zhen, Su-Jie Zhang, Jing-Ran Xu, Li Li, Xiao-Guang Zou

**Affiliations:** The First People’s Hospital of Kashgar, No.66, Yingbin Avenue, Xinjiang, Kashgar 844000 Kashgar City, China

**Keywords:** *M.tb* lineage, *M.tb* Sublineage, Branch-specific SNP, Geographical distribution, Whole-genome sequencing (WGS)

## Abstract

**Objectives:**

Kashgar prefecture is an important transportation and trade hub with a high incidence of tuberculosis. The following study analyzed the composition and differences in *Mycobacterium tuberculosis* (*M.tb*) lineage and specific tags to distinguish the lineage of the *M.tb* in Kashgar prefecture, thus providing a basis for the classification and diagnosis of tuberculosis in this area.

**Methods:**

Whole-genome sequencing (WGS) of 161 *M.tb* clinical strains was performed. The phylogenetic tree was constructed using Maximum Likelihood (ML) based on single nucleotide polymorphisms (SNPs) and verified through principal component analysis (PCA). The composition structure of *M.tb* in different regions was analyzed by combining geographic information.

**Results:**

*M.tb* clinical strains were composed of lineage 2 (73/161, 45.34%), lineage 3 (52/161, 32.30%) and lineage 4 (36/161, 22.36%). Moreover, the 3 lineages were subdivided into 11 sublineages, among which lineage 2 included lineage 2.2.2/Asia Ancestral 1 (9/73, 12.33%), lineage 2.2.1-Asia Ancestral 2 (9/73, 12.33%), lineage 2.2.1-Asia Ancestral 3 (18/73, 24.66%), and lineage 2.2.1-Modern Beijing (39/73, 53.42%). Lineage 3 included lineage 3.2 (14/52, 26.92%) and lineage 3.3 (38/52, 73.08%), while lineage 4 included lineage 4.1 (3/36, 8.33%), lineage 4.2 (2/36, 5.66%), lineage 4.4.2 (1/36, 2.78%), lineage 4.5 (28/36, 77.78%) and lineage 4.8 (2/36, 5.66%), all of which were consistent with the PCA results. One hundred thirty-six markers were proposed for discriminating known circulating strains. Reconstruction of a phylogenetic tree using the 136 SNPs resulted in a tree with the same number of delineated clades. Based on geographical location analysis, the composition of Lineage 2 in Kashgar prefecture (45.34%) was lower compared to other regions in China (54.35%-90.27%), while the composition of Lineage 3 (32.30%) was much higher than in other regions of China (0.92%-2.01%), but lower compared to the bordering Pakistan (70.40%).

**Conclusion:**

Three lineages were identified in *M.tb* clinical strains from Kashgar prefecture, with 136 branch-specific SNP. Kashgar borders with countries that have a high incidence of tuberculosis, such as Pakistan and India, which results in a large difference between the *M.tb* lineage and sublineage distribution in this region and other provinces of China.

**Supplementary Information:**

The online version contains supplementary material available at 10.1186/s12879-022-07307-4.

## Introduction

Patients affected by tuberculosis in China account for 8.4% of total tuberculosis cases worldwide (about 10 million) [1], with Xinjiang province in northwestern China being one of the most serious tuberculosis endemic areas [2]. It has been confirmed that lineage has an important role in disease prognosis, vaccine efficacy, and drug resistance [3, 4]. Accurate genotyping of *M.tb* can further the understanding of the main local epidemic strains, predict the local transmission and epidemic trend of *M.tb*, and provide strong support for the classification and diagnosis of *M.tb* clinical isolates. Compared with the traditional *M.tb* genotyping methods [5], whole-genome sequencing (WGS) can identify sequence variations at the whole genome level with higher discrimination and is more beneficial for understanding correlations among drug resistance, virulence, and tuberculosis progression. Systematic genetic relationships between the *M.tb* lineages and sublineages based on SNP have been reported in many studies. The human adaptive *M.tb* complex can be divided into 9 lineages (Lineage 1-Lineage 9); each lineage indicates diversity in different regions. Lineage 2 and Lineage 4 are widely prevalent lineages in the world [6, 7]; Lineage 5 and Lineage 6 are largely limited to West Africa [8], and Lineage 8 and lineage 9 are new lineages recently discovered in Africa [8, 9]. Coll et al. [4] suggested using single nucleotide polymorphism (SNP) as a high resolution and stable typing technique, as well as for phylogenetic and evolutionary analysis. Furthermore, 7 lineages and 55 sublineages of *M.tb* can be subdivided and denominated by 62 specific SNPs.

As Kashgar prefecture is located in an important hub of the Silk Road and borders Pakistan, India, and other countries, *M.tb* may be introduced into the region along with population migration, trade, and cultural exchange. It may also be affected by differences in host immunity and external living environment, resulting in diversity in the distribution of *M.tb* lineages and sub-lineages in different geographical regions [10–12]. At present, *M.tb* lineages in the Kashgar prefecture and the Xinjiang region are primarily detected by traditional typing methods; however, there is insufficient research on *M.tb* lineage in terms of whole-genome SNP [13].

In this study, WGS of 161 *M.tb* clinical strains in Kashgar prefecture (one city and six counties) was performed, the phylogenetic tree was constructed based on SNP information, and the specific SNP sites among lineages were selected. Combined with the source geographic information of the sample host, the *M.tb* lineage, sub-lineage composition, and genome characteristics (lineage-specific SNP) were analyzed in one city and six counties of Kashgar prefecture. Our results provided strong support for screening *M.tb* lineage/genotype in the future. Combined with the distribution of *M.tb* pedigree between Kashgar prefecture, other domestic provinces, and neighboring countries, we explored the factors affecting the differences in *M.tb* pedigree and sub pedigree distribution in Kashgar prefecture so as to provide a research basis for the diagnosis and treatment of *M.tb* in this region.

## Materials and methods

### Samples

A total of 161 *M.tb* clinical strains were collected from 2018 to 2019 in Kashgar prefecture, Xinjiang, China. The clinical strains were collected from the First People's Hospital of Kashgar, Shufu County People's Hospital, Shule County People's Hospital, Payzawat County People's Hospital, Yengisar County People's Hospital, Yarkant County People's Hospital, and Poskam County People's Hospital, which are considered the best general hospitals in their respective counties. The majority of TB patients are treated in these hospitals. The incidence rate in this area has been reported to be close to 1/10,000, which is far higher than that of 1/100,000 in other parts of China [2]. Sputum from patients' lower respiratory tracts was collected to obtain the clinical strains, and general information of each patient was acquired and sorted (Additional file 1). All patients were clinically tested for etiology, drug sensitivity, and IGRA.

Study was performed in accordance with the Declaration of Helsinki and relevant regulations (ethics approval and consent to participate). In addition, informed consent was obtained from each patient.

### Whole genome sequencing (WGS)

*M.tb* genomic DNA was extracted and purified by magnetic bead extraction kit (1,000,006,988, MGI, Shenzhen, China), and the concentration of nucleic acid was quantified by Qubit 3.0 fluorescence tool (Q33216, ThermoFisher, Shanghai, China). The qualified clinical strains were treated with MGIEasy Digesting DNA Library Preparation Kit (V2.0, MGI, Shenzhen, China) for library construction, and library fragment size was checked by an Agilent 2100 Bioanalyzer (G2939AA, Agilent Technologies, Shanghai, China). After qualified libraries were mixed, WGS was conducted on the MGI 2000 Platform (PE100, MGI, Shenzhen, China).

### Sequencing data process and mutation detection

Fastqc toolkit (V0.11.8) was used to check for the quality of the raw reads, which was followed by trimming of adapters, low-quality bases with a Phred quality score of less than 20, and fragments with large fluctuation at the beginning of each sequence. Reads shorter than 30 bp were excluded from the downstream analysis, and the effective sequence length of reads was controlled at about 80 bp. The Coverage depth of the *M.tb* genome was analyzed by the depth function of Samtools (V1.10) [14]. Samples with a coverage > 95% were selected for sequencing data. Then, reads were mapped on the reconstructed ancestral sequence of *M.tb* using Burrows-Wheeler Alignment Tool (BWA, V0.7.17) [15]. As there was no reconstruction available for an ancestral *M.tb* chromosome, the chromosome coordinates and the annotation were that of H37Rv (NC_000962.3). Duplicated reads were marked by the Mark Duplicates module of Picard (V1.119) and were excluded. SNPs were called from each alignment file using GATK (V4.0) [16]. All SNPs were annotated with H37Rv by ANNOVAR (V2.1.1) [17]. The annotation embodied the amino acid changes at the SNP site, the position information of the antigen peptide, and the gene name and Rv number.

### Phylogenetic analysis and PCA

Based on SNPs of the whole-genome sequencing (WGS) of *M.tb* clinical strains (161 cases), an ML phylogenetic tree was constructed via IQ-tree (V1.6.12) [18] using the ultrafast bootstrap (bootstrap = 1000) method. Next, KvarQ (V0.12.2) [19] was used to determine the *M.tb* complex lineage/sublineage (Additional file 3) by analyzing the spoligotyping of the sample. The phylogenetic tree was drawn and remodeled using FigTree (V1.4.4). In terms of all SNP, PCA was conducted for *M.tb* clinical strains using Plink 2.0 [20] and adegenet package of R (V4.0.5) in order to verify the accuracy of the lineages and sublineages.

### branch-specific SNPs and classification of lineages and sublineages

The dataset was split into two populations for each lineage and sublineage, one containing all samples descending the clade-defining node and the other with remaining samples. The different SNPs were obtained by comparing the two branches. To ensure that branch-specific SNPs could also be used as markers for strain typing, we adopted the following filtering criteria: (1) only synonymous mutations were retained so as to reduce selection under external pressure; (2) SNPs in the coding region were retained, due to lower frequency of insertions and deletions in the coding region; (3) the basic genes related to the growth of *M.tb* were used; (4) when comparing the differences between two branches of SNP sites, we selected the site with F-statistics (Fst) > 0.99; Fst was calculated by hierfstat of R package (value range 0 ~ 1, where 0 indicated that the two populations were random Mating; 1 indicated that the two populations were completely isolated). (5) The classification of Lineage 2, Lineage 3, Lineage 4 and its sublineages were performed according to the criteria proposed by Coll et al. [4] and Shitikov et al. [21]. The Branch-specific SNPs were selected for *M.tb* lineages and sublineages in this area (see Additional file 3 for details based on the branch-specific SNPs of *M.tb* lineage and sublineage proposed by many scholars [4, 14, 21–23]).

### Geographical distribution of lineages and sublineages

*M.tb* lineage and sublineage information were correlated with the geographical information of the 161 tuberculosis cases. The composition and differences between *M.tb* lineages and sublineages in Kashgar prefecture were analyzed and compared with those in Xinjiang's neighboring provinces (Tibet, Gansu, and Qinghai [24]), and other regions [24] in China and neighboring countries (Pakistan [25], India [26], etc.). The differences in *M.tb* lineage composition between Kashgar prefecture and the above regions were previously discussed. We downloaded China's administrative division data from the Database of Global Administrative Areas (GADM, https://gadm.org/). We then used R package sf and ggplot2 to plot the geographic distribution of Kashgar prefecture (one city and six counties) and the geographic distribution of Xinjiang's surrounding provinces, and then R Package maps to draw a geographic distribution map of China and the country bordering Xinjiang.

## Results

### Lineage and sublineage analysis of 161 *M.tb* clinical strains

**Fig. 1:**
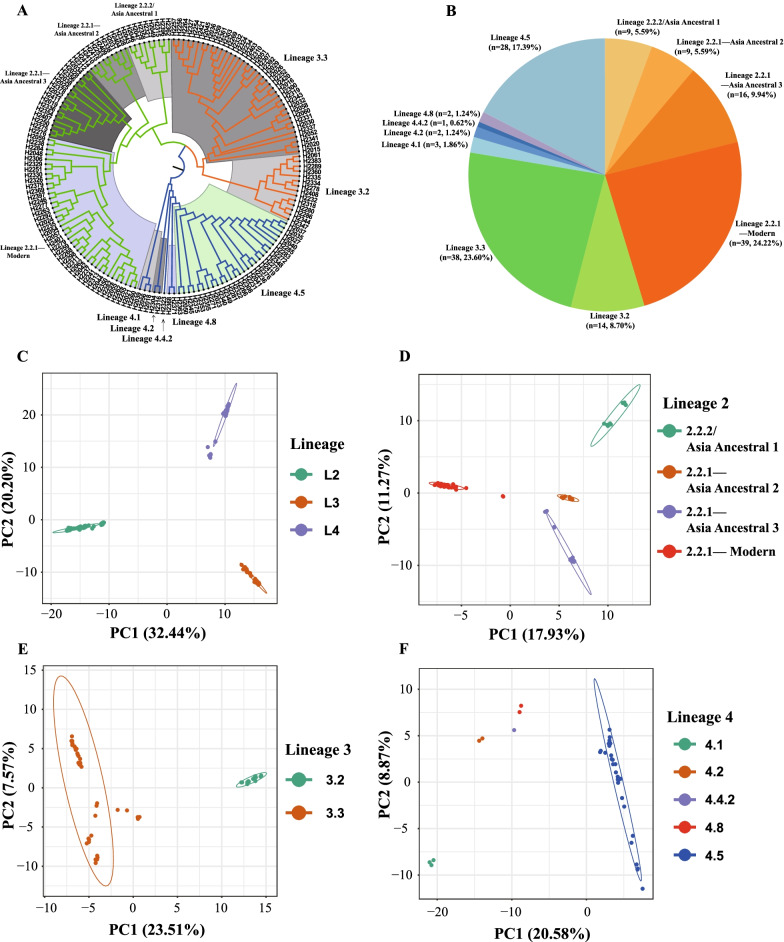
Lineage and Sublineage Analysis of 161 Cases of *M.tb* Clinical strains. **A** The phylogenetic tree of 161 cases of *M.tb* clinical strains in Kashgar prefecture (including one city and six counties) was constructed by the ML method. A total of 3 lineages (Lineage 2-Lineage 4, marked with orange, green and blue, respectively) and 11 sublineages (marked by the shaded areas) were defined, and among them, the Lineage 3 sublineage was designated in an anticlockwise order (as Lineage 3.2 and Lineage 3.3, respectively), while the N 161 cases of *M.tb* clinical strains were marked with black font. Excluding Lineage 2.2.2 sublineages, all clinical strains in *M.tb* Lineage 2 were of Lineage 2.2.1 sublineages. **B** The proportion of 9 sublineages of *M.tb* clinical strains. **C** PCA diagram of *M.tb* clinical strains of the three lineages. **D** PCA diagram of *M.tb* clinical strains in Lineage 2. **E** PCA diagram of *M.tb* clinical strains in Lineage 3. **F** PCA diagram of *M.tb* clinical strains in Lineage 4

Based on 21,438 SNPs, 161 *M.tb* clinical strains in Kashgar prefecture (including one city and six counties) were divided into three main branches using the phylogenetic tree constructed via the ML method (Fig. 1A). One branch sample was clustered with corresponding lineage reference strains, namely Lineage 2 (73/161, 45.34%), Lineage 3 (52/161, 32.30%), and Lineage 4 (36/161, 22.36%). Three lineages were further divided into 11 sublineages according to branch-specific SNPs (Fig. 1A). Seventy-three *M.tb* of lineage 2 were entirely from Lineage 2.2, of which 64 *M.tb* were Lineage 2.2.1 (87.67%), and the other 9 *M.tb* were Lineage 2.2.2/Asia Ancestral 1 (12.33%). Lineage 2.2.1 was further divided into 3 sublineages, corresponding to Asia Ancestral 2 (9/73, 12.33%), Asia Ancestral 3 (16/73, 21.92%), and the Modern Beijing sublineage (39/73, 53.42%), respectively. Fifty-two *M.tb* of Lineage 3 were divided into two main branches in the phylogenetic tree without Lineage 3.1 sublineage. Therefore, Lineage 3.2 (14/52, 26.92%) and Lineage 3.3 (38/52, 73.08%) were named anticlockwise in the phylogenetic tree, respectively. Thirty six *M.tb* of Lineage 4 were divided into 5 sublineages, corresponding to Lineage 4.1 (3/36, 8.33%), Lineage 4.2 (2/36, 5.66%), Lineage 4.4.2 (1/36, 2.78%), Lineage 4.5 (28/36, 77.78%) and Lineage 4.8 (2/36, 5.66%), respectively. Among the 9 mentioned sublineages, Lineage 2.2.1-Modern Beijing sublineages (39/161, 24.22%), Lineage 3.3 (38/161, 23.60%), and Lineage 4.5 (28/161, 17.39%) had the highest proportion in each lineage, which were the main epidemic strains in Kashgar prefecture (Fig. 1B). PCA results were consistent with the above findings (Fig. 1C). The results showed that the samples could be divided into three main lineages, namely L2, L3, and L4. Among them, PC1 and PC2 were the most important and could cumulatively explain 52.64% (Fig. 1C). PCA of the three main lineages was further performed, and the sublineages were divided (Fig. 1D–F).

### Specific SNPs of 161 *M.tb* clinical strains

One hundred thirty-six branch-specific SNPs were obtained by screening. Reconstruction of a phylogenetic tree using the 136 SNPs for all 161 samples resulted in a tree with the same number of delineated clades (Additional file 2). The branch-specific SNPs of each lineage and sublineage are shown in Table 1 and Additional file 3. There were 14 branch-specific SNPs in Lineage 2, among which 9 SNPs have not been reported before. In addition, 12 branch-specific SNPs were screened in 4 sublineages (Lineage 2.2.2/Asia Ancestral 1, Lineage 2.2.1-Asia Ancestral 2, Lineage 2.2.1-Asia Ancestral 3 and Lineage2.2.1-Modern Beijing sub-lineage were 6, 4, 1, and 1, respectively), and these branch-specific SNPs of *M.tb* sublineages were found for the first time. There were 14 branch-specific SNPs in Lineage 3, among which 9 SNPs were different from the reported SNPs. In addition, there were also three branch-specific SNPs in sublineages 3.2 and sublineages 3.3. There were 10 branch-specific SNPs in Lineage 4, among which 7 SNPs have not been reported before. In addition, 72 branch-specific SNPs were screened in 5 sublineages (Lineage 4.1, Lineage 4.2, Lineage 4.4.2, Lineage 4.5, and Lineage 4.8 were 18, 18, 20, 13, and 3, respectively), and the branch-specific SNPs of *M.tb* strain were found for the first time. The sublineage branch-specific SNPs were the same as those in Coll’s report. To summarize, branch-specific SNPs were found for 161 *M.tb* clinical strains in Kashgar prefecture (including one city and six counties).

**Table 1:** Specific SNP of 3 Lineages and 11 Sublineages in this Study

Lineage	Sublineage			Name	N	Number of specific	Number of specific	Common SNP
						SNP	SNPs in Coll’s study	
2				East Asian	73	14	6	5(5/14, 35.71%)
	2.1			Protobeijing	–	–	12	–
	2.2			Beijing 2.2	–	–	5	–
		2.2.1		Beijing 2.2.1	64	8	2	2(2/8, 25%)
			*	Asia Ancestral 2	9	4	–	–
			*	Asia Ancestral 3	16	1	–	–
			*	Modern Beijing	39	1	–	–
		2.2.2		Asia Ancestral 1	9	6	6	5 (5/6, 83.33%)
3				India and East Africa	52	14	9	5 (5/14, 35.71%)
	3.1				–	–	–	–
		3.1.1			–	–	3	–
		3.1.2			–	–	2	–
	3.2				14	3	–	–
	3.3				38	3	–	–
4				Euro-American	36	10	3	3 (3/10, 30.00%)
	4.1				3	18	1	1 (1/18, 5.56%)
	4.2				2	18	8	8 (8/18, 44.44%)
	4.4				–	–	2	–
		4.4.2			1	20	11	11 (11/20, 55.00%)
	4.5				28	13	8	6 (6/13, 46.15%)
	4.8				2	3	1	1 (1/3, 33.33%)

Number of specific SNP referred to SNP with *M.tb* mostly appearing in 3 lineages and 11 sublineages. Common SNP indicated the same specific SNP reported by Coll et al*.* [4]

The evolutionary tree shows that the lineage marked with “*” belongs to the branch of Beijing lineage 2.2.1, but it has not been marked in previous studies (such as 2.2.1.1, 2.2.1.2, 2.2.1.3)

### Geographical distribution of lineages/sublineages

**Fig. 2:**
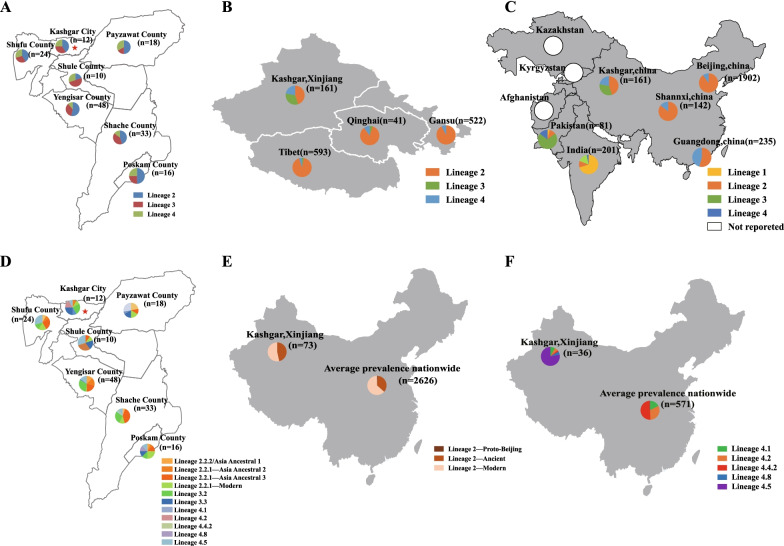
Comparison and Geographical Distribution of *M.tb* Lineages and sublineages. **A** Lineage composition and distribution of 161 cases of *M.tb* Lineage 2-Lineage 4 in Kashgar prefecture (including one city and six counties). **B** *M.tb* Lineage composition of Kashgar prefecture and adjacent provinces (Tibet, Qinghai, and Gansu Provinces). **C** *M.tb* Lineage composition of Kashgar prefecture, some domestic provinces and cities (Shanxi province, Beijing, and Guangdong province), and neighboring countries (Kazakhstan, Kyrgyzstan, Tajikistan, Afghanistan, Pakistan, and India). There was no report on *M.tb* lineages in Kazakhstan, Kyrgyzstan, Tajikistan, and Afghanistan, which are respectively marked with white circles in **C**. **D** Sublineage composition and distribution of 161 cases of *M.tb* Lineage 2-Lineage 4 in Kashgar prefecture. **E** The proportion of *M.tb* Lineage 2 sublineages in Kashgar prefecture and the whole country. **F** The proportion of *M.tb* Lineage 4 sublineages in Kashgar prefecture and the whole country

*M.tb* lineage/sublineage information was correlated with the geographical information of tuberculosis patients (Fig. 2A). *M.tb* clinical strains of Kashgar prefecture were composed of 3 lineages (Lineage 2, Lineage 3, and Lineage 4). The proportion of *M.tb* lineages in the six Kashgar prefecture counties was similar. Compared with neighboring provinces such as Tibet, Qinghai, and Gansu (Lineage 2: 85.00%–92.12%, Lineage 3: 0.20%–5.00%, and Lineage 4: 4.98%–10.00%), Kashgar prefecture had a lower proportion of Lineage 2 (45.34%) and a higher proportion of Lineage 3 (32.30%) and Lineage 4 (22.36%) (Fig. 2B). In Kashgar prefecture, the proportion of Lineage 2 (45.34%) was lower than the national average (70.00%), the proportion of Lineage 3 was higher than the national average (0.92%–2.01%), and the proportion of Lineage 4 was similar to the national average (24.82%–25.25%) (Fig. 2C). Compared with neighboring countries, the main *M.tb* epidemic lineages in Pakistan and India were Lineage 3 (70.40%) and Lineage 1 (70.15%), respectively. The proportion of Lineage 3 (32.30%) was relatively low, and there was no Lineage 1 in Kashgar prefecture (Fig. 2C). *M.tb* lineage in Kashgar prefecture was more complex compared to other regions of China, there are some lineages unique to surrounding countries. Thus, it is speculated that the Lineage 3 strains could be introduced from neighboring countries in Kashgar prefecture.

The Lineage2.2.1-Modern Beijing sublineage of Lineage 2 was distributed in all counties except Shule county, which had the highest proportion (33.33%–86.67%) of the Lineage 2 in Kashgar prefecture (Fig. 2D). The Lineage2.2.1-Modern Beijing sublineage (57.45%) was also predominant in other regions of China (Fig. 2E). Except for Poskam County, the proportion of Lineage 3.3 (42.86%–93.75%) was higher than that of Lineage 3.2 (6.25%–57.14%) in all other cities and counties in this region. Moreover, the Lineage 3.1 sublineage was predominant in other regions of China (Fig. 2D). As for Lineage 4, the Lineage 4.5 sublineage was the dominant sublineage, which had the highest proportion (33.33%–100%) in each city and county in Kashgar prefecture (including one city and six counties) (Fig. 2D). Additionally, 3 Lineage 4.1 strains were found in Payzawat, Yengisar, and Shache County, respectively; 2 Lineage 4.2 strains were found in Kashgar city; 1 Lineage 4.4.2 strain was found in Shache County; 2 Lineage 4.8 strains were found in Poskam County; the remaining 28 *M.tb* were all Lineage 4.5 strains. Lineage 4 was mainly composed of Lineage 4.2 (7.78%), Lineage 4.4 (31.11%), and Lineage 4.5 (60.00%) in other regions in China (Fig. 2F). In brief, the Lineage 2.2.1-Modern Beijing sublineage and Lineage 4.5 were the primary dominant *M.tb* strains in other regions in China, and the sublineage composition of Lineage 3 was different in other regions in China compared to Kashgar prefecture.

## Discussion

Kashgar prefecture is located in Northwest China. It is a crucial transportation hub for cultural exchange, tourism, and economic trade between China and other countries in Central Asia. China has the second-largest tuberculosis epidemic, with more than 1.3 million new cases every year [27]. Since Kashgar prefecture borders Pakistan, India, and other countries with high tuberculosis rates, it may impact the local *M.tb* distribution. Also, there are vast differences in the pathogenicity of *M.tb* across different lineages [27, 28]. Understanding the distribution of *M.tb* lineages is beneficial for classifying and diagnosing *M.tb* clinical isolates.

In this study, 161 cases of *M.tb* clinical strains were composed of Lineage 2, Lineage 3, and Lineage 4, which is consistent with Chen H ‘s results on the distribution of *M.tb* isolates in the Xinjiang region [29]. This study confirmed that Lineage 2 was dominant in all provinces in China (the national average was 70%, and the proportion in Xinjiang was only 44%) [24, 30], and the composition of Lineage 2 (73/161, 45.34%) in Kashgar prefecture was lower than the national average and consistent with the above results. In the present study, Lineage 3 was spread through the areas constituting Silk Road [30] and was concentrated in northwestern China. A total of 62% of Lineage 3 (CAS/Delhi) strains in China were found in Xinjiang [31]. Lineage 3 strains were also found in provinces and cities adjacent to Xinjiang (Tibet and Qinghai Provinces), and the composition of Lineage 3 was higher than in other prefectures [24]. In addition, Lineage 3 (70.40%) was dominant in Pakistan, a country with a high tuberculosis rate bordering Kashgar prefecture [25]. In this study, Lineage 3 in Kashgar prefecture accounted for 32.30%, which was much higher than in surrounding provinces (0.20%–5.00%) and other provinces in China (0.92%–2.01%) but was similar to neighboring countries. As Kashgar prefecture borders Pakistan, and the border crossing (Khunjerab Port) is located there, It is speculated that the frequent movement of people between the two places caused the spread of Lineage 3 strains to other provinces. Lineage 4 is highly prevalent in western China [29, 32] and mainly consists of the Lineage 4.5 sublineage (primarily in Xinjiang for the geographical restriction), while it is absent in the Americas and Africa [33]. In this study, in regards to Lineage 4 (36/161, 22.36%), the Lineage 4.5 sublineage (28/36, 77.78%) was also prevalent in Kashgar prefecture with a higher proportion than that of other regions in China, which is consistent with the above study. Xinjiang is located in the middle of Eurasia and is a crucial transportation hub for the Silk Road Economic Belt. It is possible that lineage 4.5 spread to the region through the ancient Silk Road, which led to the prevalence of this sublineage in the area.

WGS can rapidly provide genotypes and drug-resistant types for epidemiological surveillance. Coll et al. [4] proposed the classification markers for 7 lineages of *M.tb* and their sublineages based on 1601 *M.tb* genomic data analysis. *M.tb* clinical strains could be accurately classified based on these markers. Prasit and colleagues [34] classified 480 cases of Lineage 1 clinical strains in Thailand into 18 sublineages based on the markers mentioned above. In this study, 3 Lineages of *M.tb* clinical strains were further divided into 11 sublineages based on the reported specific SNP [4, 21–23]. After screening, 136 branch-specific SNPs were obtained, among which 89 SNPs were different from those reported by Coll et al. [4]. Furthermore, the newly identified Lineage 3.2 and Lineage 3.3 may be specific sublineages in Kashgar prefecture, and their specific classifications should be further investigated in future studies. The above results further elucidate *M.tb* lineage and sublineage marker SNP and provide strong support for the classification and diagnosis of *M.tb* clinical isolates. They also further the understanding of *M.tb*-specific SNP in Kashgar prefecture.

Our data show that tuberculosis in China in most patients is caused by *M.tb* Lineage 2 and Lineage 4 [24], which are both more pathogenic than other lineage isolates [28]. Also, there may be a possible correlation between Lineage 4 and non-Han populations [35] and between Lineage 3 and the Uygur nationality [29]. In the past, Kashgar prefecture was a crucial transportation hub for the ancient Silk Road, while today, it is an international trading port where Chinese and foreign merchants gather. In addition, it borders Pakistan, India, and other countries that are frequently affected by tuberculosis and are characterized by frequent movement from people from neighboring countries, which may be one of the key reasons for the different proportion of *M.tb* lineage between Kashgar prefecture and other regions in China.

To sum up, we studied the composition and difference of *Mycobacterium tuberculosis* (*M.tb*) lineage in the area through high-throughput sequencing. One hundred and sixty-one *M.tb* clinical strains from Kashgar prefecture (including one city and six counties) were divided into 3 lineages and 11 sublineages, with region-specific SNP. Considering the geographical distribution of *M.tb*, it was found that the composition of *M.tb* lineage in Kashgar prefecture was more complex than in other regions in China, and the proportion of *M.tb* lineage in Kashgar City was different from the other six counties in this region. Lineage 3 was the main prevalent strain in Pakistan, but it was only prevalent in the Xinjiang region in China, which may explain why this lineage strain have spread from neighboring countries. Our data provided a fundamental basis for the study of *M.tb* lineages and the classification and diagnosis of tuberculosis in Kashgar prefecture.

## Supplementary Information

**Additional file 1.** Samples information.

**Additional file 2.** Phylogenetic tree based on 136 branch-specific SNPs.

**Additional file 3.** 136 branch-specific SNPs in *M.tb.*

## Data Availability

All data generated or analyzed during this study are included in this published article [and its additional information files]. The datasets generated and analyzed during the current study are available from the NGDC database (https://ngdc.cncb.ac.cn/). The assigned accession of the submission is CRA005180. The data can be found at the following links https://bigd.big.ac.cn/gsa/browse/CRA005180.
