# Supplementary material for: Distribution and identification of Mycobacterium tuberculosis lineage in Kashgar prefecture
Source: BMC Infect Dis. 2022 Mar 30;22:312. doi: 10.1186/s12879-022-07307-4 (PMC8966310; doi:10.1186/s12879-022-07307-4)
Supplement: Supplementary file 2 — Additional file 2. Phylogenetic tree based on 136 branch-specific SNPs. [file 12879_2022_7307_MOESM2_ESM.pdf]

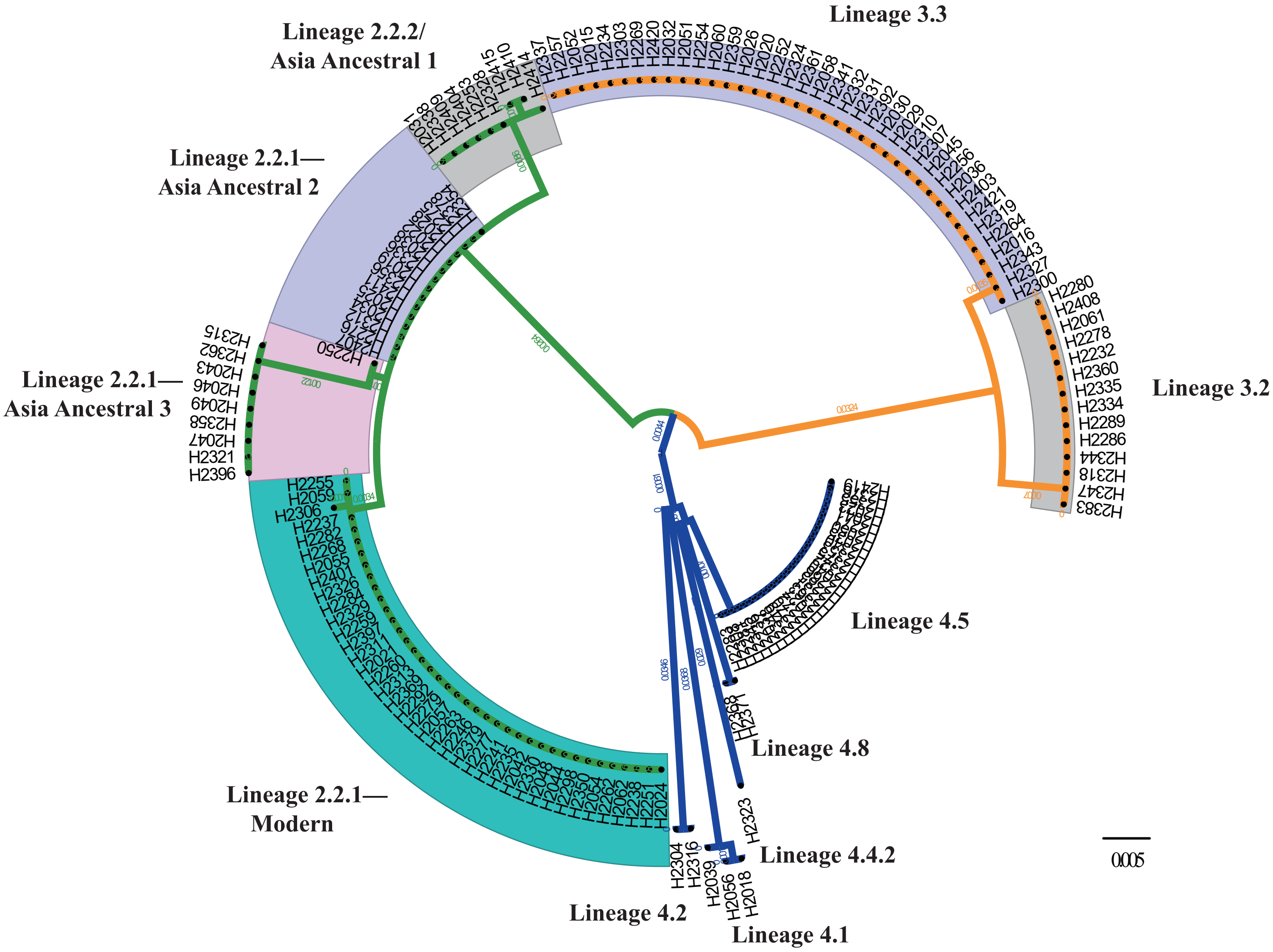

Additional file 2. Phylogenetic tree based on 136 Branch-Specific SNPs
